# Non-Covalent Synthesis of Metal Oxide Nanoparticle–Heparin Hybrid Systems: A New Approach to Bioactive Nanoparticles

**DOI:** 10.3390/ijms140713463

**Published:** 2013-06-27

**Authors:** Elena Vismara, Antonio Valerio, Alessia Coletti, Giangiacomo Torri, Sabrina Bertini, Giorgio Eisele, Rosalba Gornati, Giovanni Bernardini

**Affiliations:** 1 Department of Chemistry, Materials and Chemical Engineering “G. Natta” Polytechnic, 7 Mancinelli Street, 20131 Milan, Italy; E-Mails: avalerio@chem.polimi.it (A.V.); alessia.coletti@chem.polimi.it (A.C.); 2 Interuniversity Center “The Protein Factory,” Polytechnic of Milan, ICRM-CNR Milan and Insubria University, 21100 Varese, Italy; 3 Ronzoni Institute for Chemical and Biochemical Research, 81 G. Colombo Street, 20133 Milan, Italy; E-Mails: torri@ronzoni.it (G.T.); bertini@ronzoni.it (S.B.); eisele_cat@ronzoni.it (G.E.); 4 Department of Biotechnology and Molecular Sciences, University of Insubria, 3 Dunant Street, 21100 Varese, Italy; E-Mails: rosalba.gornati@uninsubria.it (R.G.); giovanni.bernardini@uninsubria.it (G.B.)

**Keywords:** heparin, transition metal oxide nanoparticles, organic inorganic nanostructures, electrostatic interaction, NMR spectroscopy, microscopy

## Abstract

Heparin has been conjugated to Fe_3_O_4_, Co_3_O_4_, and NiO nanoparticles (NPs) through electrostatic interactions, producing colloidal suspensions of hybrid metal oxide heparin NPs that are stable in water. Negative zeta potentials and retention of heparin’s ability to capture toluidine blue indicate that heparin’s negative charges are exposed on the surface of the coated NPs. IR results confirmed the formation of nanohybrids as did NMR experiments, which were also interpreted on the basis of toluidine blue tests. Transmission electron microscopy results revealed that the heparin coating does not modify the shape or dimension of the NPs. Dynamic light scattering and negative zeta potential measurements confirmed that heparin surface functionalisation is an effective strategy to prevent NP aggregation.

## 1. Introduction

Nanoparticles (NPs) can act as core materials, providing a useful platform for surface functionalisation, for applications in biomedicine, catalysis, and electronic devices [1]. The use of composite NPs in analytical chemistry has also been specifically reviewed [2].

Since the early 2000s, much effort has been made to combine the intrinsic properties of biomolecules with the unusual and size-dependent material properties of NPs [3]. When integrated with biomolecules, NPs with metallic, metal oxide (MO), semiconductor, or silica cores have been used in a wide range of applications in biology and medicine [4,5]. Moreover, Au NPs are emerging as therapeutics in their own right, with the capability of modulating cellular processes based on their surface functionality [6].

A recent review summarised the synthesis and biofunctionalisation of inorganic metal, semiconductor and magnetic nanoparticles for applications in many areas of biomedicine, from diagnostics to the treatment of diseases [7]. The rapid detection of Hendra virus using magnetic particles and quantum dots has also been reported recently [8].

A special issue on theranostic nanomedicine [9] listed twenty-eight accounts of various inorganic NPs, lipid aggregates and synthetic polymer systems, detailing the physicochemical properties of these biocompatible NP platforms. Unfortunately, however, the very active research on new nanomaterials that are potentially useful in medicine has not been counterbalanced by an adequate knowledge of their pharmacokinetics and toxicity [10].

In this study, we describe how to engineer transition metal oxide NPs of the three ferromagnetic elements, Fe, Co and Ni (*i.e.*, Fe_3_O_4_, Co_3_O_4_, and NiO) with heparin, with the aim of merging the properties of the individual components.

The natural occurring anticoagulant heparin is a heterogeneous, polydisperse, highly sulphated glycosaminoglycan composed of 1→4 linked disaccharide repeating units, which consist of an α-d-glucosamine (A) and a hexuronic acid, α-l-iduronic (I) or β-d-glucuronic (G) acid unit, with *O*-sulphate groups at different positions on the disaccharide unit [11]. The most frequently occurring repeating disaccharide sequence is α-l-iduronic acid-2-*O*-sulphate-α-D-glucosamine-*N*,6-disulphate (I2S-ANS,6S), which represents the highly sulphated segment of heparin, whereas under-sulphated sequences contain ANAc (α-d-glucosamine-*N*-acetyl) and non-sulphated, I and G. Heparin and low molecular weight heparin have both been recognised as anti-angiogenic agents and vectors that are able to bind proteins that are involved and over-expressed in the tumour microenvironment [12,13]. Some chemically modified heparin species have also been reported to exert a beneficial effect in cancer patients, regardless of the mode of action [14].

Iron oxide (Fe_3_O_4_) and Fe-based alloy NPs, such as iron-cobalt (FeCo), provide a unique nanoplatform for theranostic applications because of their biocompatibility, their responses to external magnetic fields, and their sizes, which are comparable to those of functional biomolecules [15]. Furthermore, because of the ease of fabrication and their approved clinical usage, Fe_3_O_4_ NPs with controlled sizes and controlled surface chemistry have been studied extensively for Magnetic Resonance Imaging (MRI) and Magnetic Fluid Hyperthermia (MFH) applications [16,17]. Superparamagnetic Iron Oxide NPs (SPION) were recently introduced for MRI, drug delivery, drug-targeting, and hyperthermia through intra- or extra-venous injection [18–20]. Surface engineering of iron oxide NPs has been widely studied for targeted cancer therapy [21].

As reported by Klostergaard [22], based on the advanced stage of preclinical human tumour xenograft studies with Fe-based magnetically responsive nanoparticles, there is great confidence that the broader requirements for moving forward to clinical applicability will be met. Therefore, our interest focuses on Fe_3_O_4_.

The same paper also investigated Co_3_O_4_ and NiO and discussed their toxicity and the risk of their use. Although we are conscious of the toxicity aspects of administering NPs [23–25], the many applications of Co_3_O_4_ and NiO nanohybrids prompted us to prepare and characterise metal oxide-heparin nanohybrids. The presence of cobalt and nickel within biofunctional magnetic NPs for the manipulation of proteins and cells supported our challenge [26].

On the nanoscale, Co_3_O_4_ exhibits magnetic, optical, field emission and electrochemical properties that are attractive in device applications [27–29]. Cobalt ferrite (mixed cobalt iron oxide) NP formation was studied in the presence of a synthetic polypeptide, and coating this material with histidine induced cellular biocompatibility [30,31]. NiO can form part of electrochemical devices, in which Ni ions are also used as an agent for chelating biomolecules [32,33]. A nonenzymatic glucose sensor based on a nickel(II)oxide/ordered mesoporous carbon-modified glassy carbon electrode has also been recently reported [34].

Concerning polysaccharides, dextran-coated iron oxide NPs have been proposed as a versatile platform for targeted molecular imaging, molecular diagnostics, and therapy [35].

In addition, Chertok *et al.* [36] argued clearly how and why iron oxide NPs could be promising drug delivery vehicles for MRI-monitored magnetic targeting of brain tumours. Currently, we focus on a heparin skeleton with the aim of taking advantage of the multiple roles that heparin can play. In particular, the heparin coating could specifically direct iron oxide NPs to a tumour mass microenvironment, where their paramagnetic properties can be simultaneously exploited for MRI and hyperthermia, and could exert non-conventional drug activity inside the tumour cells because of the ability of the NPs to cross the cell membrane.

## 2. Results and Discussion

Complex nanostructures of inorganic elements coated by polymers have been covalently linked to heparin through spacers [37,38]. Covalent linkages between nanomaterials and biomolecules often lead to a reduction in the biological activity of the biomolecules once attached to the nanoparticle’s surface [39,40]. Thus, non-covalent bioconjugation techniques, based on hydrogen bonding, electrostatic interactions and antibody–antigen interactions, are often advantageous for the biofunctionalisation of nanomaterials [41,42].

In this work, the creation of MO@heparin NPs was achieved via the electrostatic attachment of heparin to the surface of NPs. Because heparin was not manipulated, its structure in MO@heparin NPs was reasonably preserved. Another example of non-covalent heparin bioconjugation on poly-l-lysine-coated iron oxide NPs, [43] has been published, to which we refer in detail in the discussion.

MO@heparin NPs were prepared, as detailed in the experimental section, using a one-pot coating reaction at room temperature; by this method, the formation of any by-products was avoided, and the excess of heparin as a starting material could be easily recovered and recycled. The washing procedure with diethyl ether removed water and residual non-linked heparin and provided the final MO@heparin NPs which were characterised using a colorimetric assay, FT-IR spectroscopy, DLS and zeta potential measurements, TEM and ^1^H NMR spectroscopy.

The NP weight increase observed after coating (Table 1) indicates the order of magnitude of the heparin:metal ratio.

**Figure 1:**
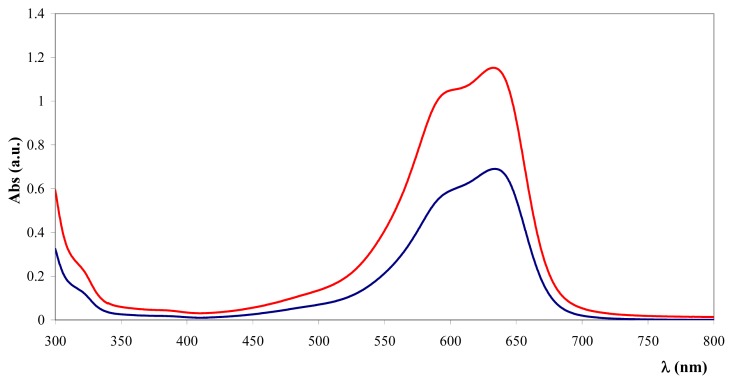
UV-Vis absorption spectra of TB before ( 
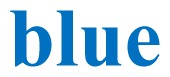
) and after ( 
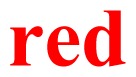
) the addition of Fe_3_O_4_@heparin NP. The comparison highlights the depletion of TB in the supernatant upon the addition of Fe_3_O_4_@heparin NP.

Gravimetric data can sometimes be underestimated because of the difficulty of recovering and handling the wet material. Therefore, the toluidine blue (TB) colorimetric method, used to assay the heparin content in immobilised heparin preparations, was tested on heparin-coated NPs [44]. This method is based on the metachromatic effect induced by heparin sulphate groups on TB in the 400 to 650 nm wavelength range. All of the coated NPs were effective at inducing the metachromatic effect. The same experiment performed with the addition of bare NPs did not influence the TB absorbance. Figure 1 reveals the spectral characteristics of TB supernatant depletion upon addition of Fe_3_O_4_@heparin NP.

Based on a standard curve reported in the experimental section, the amount of coating for 100 mg of NPs was measured (Table 1).

Co_3_O_4_@heparin and NiO@heparin NPs were repeatedly analysed by TB assay. The data range reported in Table 1 fit well with the gravimetric data. The Fe_3_O_4_@heparin NP colorimetric data range appears a little narrower and the gravimetric datum seems underestimated. Fe_3_O_4_@heparin NP was prepared several times and their TB assay appears quite reproducible.

By averaging the gravimetric and colorimetric data in Table 1, the amount of immobilised heparin can be reasonably evaluated to be in the range of 10%–20% (*w:w*) when heparin is in large excess. Decreasing the amount of heparin during the synthesis, as for Fe_3_O_4_@heparin NPs (Table 2), results in a lower functionalisation of Fe_3_O_4_.

**Figure 2:**
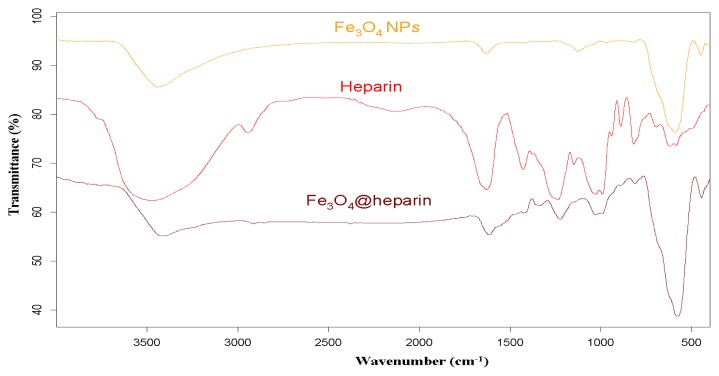
FT-IR spectra of Fe_3_O_4_@heparin NP compared with those of heparin and bare Fe_3_O_4_ NP.

**Figure 3:**
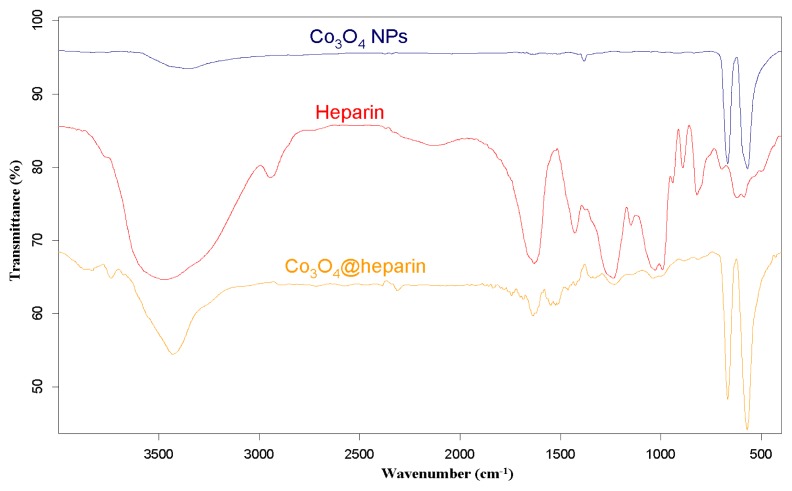
FT-IR spectra of Co_3_O_4_@heparin NP compared with those of heparin and bare Co_3_O_4_ NP.

**Figure 4:**
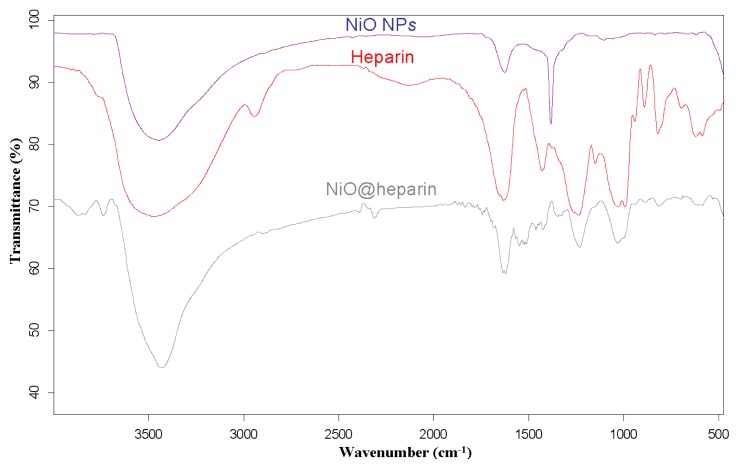
FT-IR spectra of NiO@heparin NP compared with those of heparin and bare NiO NP.

For each MO@heparin NP preparation, the presence of heparin was detected by FT-IR spectra. FT-IR spectra of NPs before and after modification with heparin are presented in Figures 2–4, along with the reference spectra for heparin.

In the NP spectra, two bands characteristic of adsorbed water (1631 cm^−1^) and carbonate stretching (1384 cm^−1^) are observed, resulting from the exposure of the samples to air [45,46]. A broad OH stretching band of hydrogen-bonded water is observed at 3438 cm^−1^. The two broad bands at 1631 cm^−1^ from moisture and at 1125 cm^−1^ exhibited by Fe_3_O_4_ are quite similar to those observed for the unmodified iron oxide sample by Khurshid [43]. In the range 900–400 cm^−1^, the IR bands of solids are usually assigned to the vibration of ions in the crystal lattice [47]. The spectra of pure solids consisted of two bands in the range 592–499 cm^−1^ for Fe_3_O_4_ and in the range 667–568 cm^−1^ for Co_3_O_4_, whereas one band appeared at approximately 438 cm^−1^ for NiO.

In the FT-IR spectra of the MO@heparins NPs, the main bands of heparin are clearly distinguishable. After coating the particles with heparin, the sulphate bands of heparin at 1240–1260 cm^−1^ and 1019 cm^−1^ were clearly visible, confirming the presence of heparin on MO@heparin NPs. Some bands in the range 1550–1400 cm^−1^ can be assigned to CO_2_^−^ stretching. In MO@heparin NPs, the sulphonamide –NHSO_3_^−^ stretching bands at 891 cm^−1^ are of lower intensity than in heparin, in perfect agreement with the spectra of heparin-coated Fe_3_O_4_ described by Khurshid [43].

**Figure 5:**
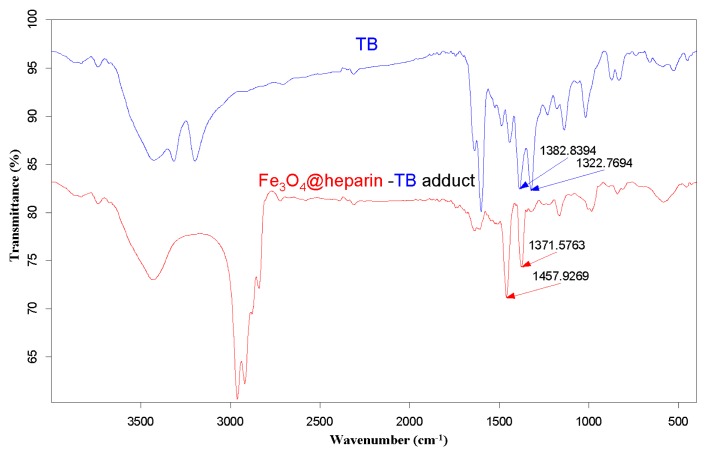
FT-IR spectra of the Fe_3_O_4_@heparin-TB adduct ( 
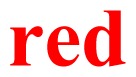
) and TB ( 
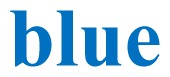
).

**Figure 6:**
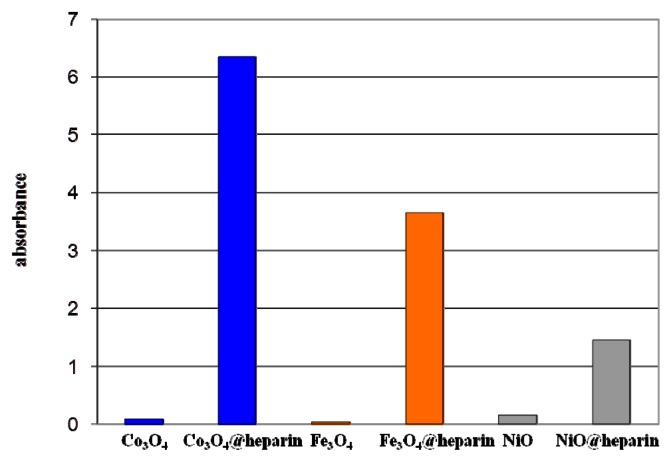
Turbidity effect for MO@heparin NP and bare NP suspensions.

The purple centrifuged MO@heparin-TB adducts were dried under vacuum and were also investigated using IR. The FT-IR spectra in Figure 5 demonstrate that the stretching frequencies of the TB functional group -N^+^(CH_3_)_2_ shifted from 1382.84 cm^−1^ and 1322.77 cm^−1^ (free TB) to 1457–1460 cm^−1^ and 1371–1377 cm^−1^ (TB interacting with heparin). These shifts are consistent with the structure of the MO@heparin-TB adducts, in which heparin’s negative charges are associated with TB’s positive quaternary amino group -N^+^(CH_3_)_2_. Figure 6 presents a comparison between TB and the Fe_3_O_4_@heparin-TB adduct. The bare NPs that were added to the TB solution were black in color when recovered and did not yield significant IR spectra.

Figure 6 reports light absorption at 600 nm for the coated and bare NPs suspended in distilled water (1 mg/mL). The coated NP suspensions reach higher absorbance values (NiO@heparin < Fe_3_O_4_@heparin < Co_3_O_4_@heparin). This effect is permanent, as the coated NP suspensions remained unaltered.

Dynamic light scattering (DLS) experiments produced further information about the supernatant of the MO@heparin NPs water suspensions. For samples described in Table 1, more than 50% of NiO@heparin NP preparation and Co_3_O_4_@heparin NP preparation was solubilised, whereas only approximately 20% of the Fe_3_O_4_@heparin NP preparation was solubilised.

**Figure 7:**
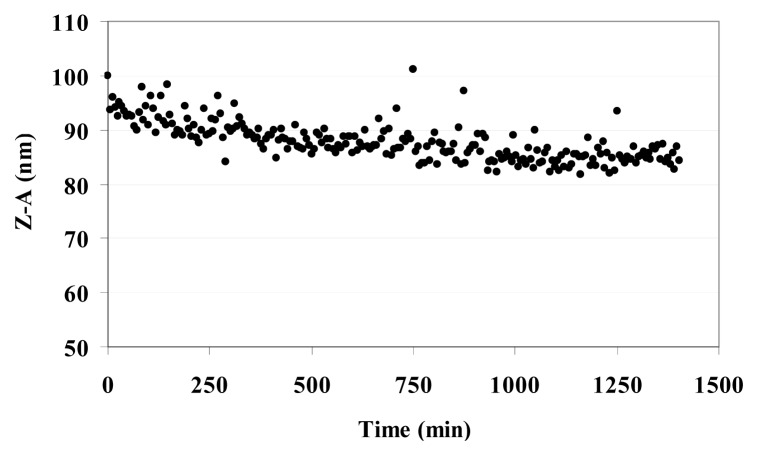
Kinetic behaviour of Fe_3_O_4_@heparin NP in H_2_O at 37 °C.

Table 3 reports the Z-Average (Z-A) value as the mean value of the hydrodynamic radius of suspended MO@heparin NPs and the PolyDispersity Index (PDI) which is a measure of the width of the NP size distribution. The Z-A for all three MO@heparin NPs appear to be quite similar, with PDI values <0.3; in contrast, DLS experiments on bare NPs showed a dispersion of larger NPs that rapidly aggregate, yielding a high PDI (data not shown). The lower aggregation of MO@heparin NPs is a result of the presence of the heparin coating. The behaviour of a suspension of Fe_3_O_4_@heparin NP in water was followed for 24 h. The Z-A tended to a constant value in 12 h (Figure 7).

The ζ measurements of bare NPs and of coated NPs described in Table 1 were performed at a concentration of 25 μg/mL in water and recorded at 25 °C, see Table 4. The heparin coating resulted in MO@heparin NPs having a highly negative surface charge, consistent with the colorimetric assays in the presence of TB. The same trend has been observed for non-covalent bioconjugation of heparin to poly-l-lysine-coated iron oxide NPs [43].

The size and the surface charge of Fe_3_O_4_@heparin NP were also measured in the presence of free heparin (Table 5). The addition of different amounts of heparin to a solution of Fe_3_O_4_@heparin in water did not result in any change in its Z-A and PDI values (as determined by DLS measurements). Fe_3_O_4_@heparin NP maintained the highly negative surface charge as shown by ζ measurements. Before running the analyses, all the samples were left for 1 h to reach equilibrium. This delay could explain the difference of Z-A between 92 and 118 and of the PDI between 0.17 and 0.24 for the starting sample, see Table 3.

Fe_3_O_4_@heparin NPs were also prepared in PBS and in the presence of the surfactant Tween 20^®^, as detailed in the experimental section. The heparin coatings are quite the same as for the Fe_3_O_4_@heparin NP synthesised in water (see Table 6). The PDI values are, however, much higher than those measured for the Fe_3_O_4_@heparin NP synthesised in water (see Table 3). The Z-A calculation makes sense only for the Fe_3_O_4_@heparin NP prepared in the presence of Tween 20^®^. ζ values put in evidence a negative surface charge for all the samples. During the pre-treatment for DLS analysis, a large portion of the Fe_3_O_4_@heparin NP prepared in PBS was solubilised in water. The coating in PBS seems interesting as it could make coated NPs soluble in water.

**Figure 8:**
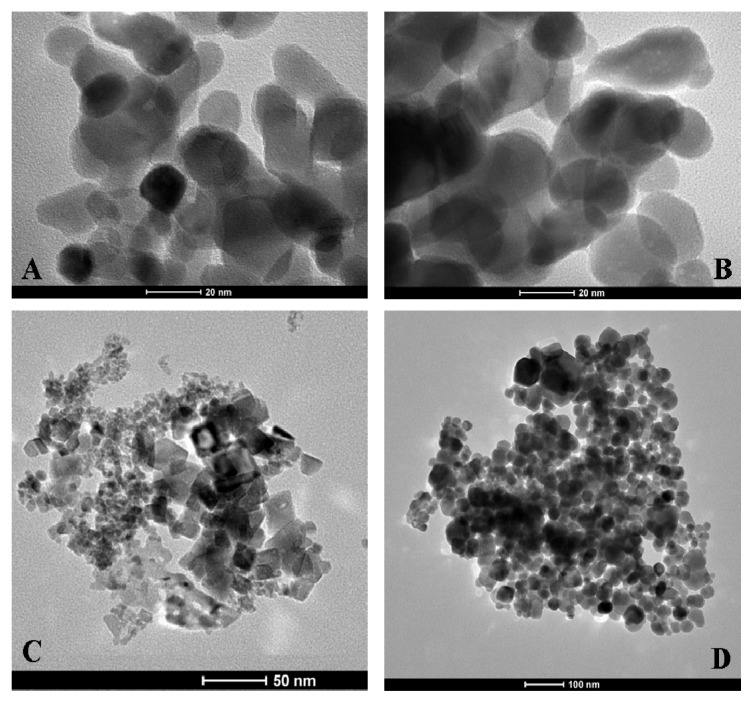
Transmission electron microscopy of MO NPs. **Panels A** and **B** reveal Co_3_O_4_ and Co_3_O_4_@heparin NPs. **Panel C** demonstrates the large heterogeneity in the size and shape of the NiO NPs. Fe_3_O_4_ NPs are shown in **panel D**.

Transmission electron microscopy (TEM) images of coated and uncoated NPs are presented in Figure 8. As expected, electron microscopy does not reveal any apparent differences between the coated and uncoated NPs (Figure 8A,B). This finding is possibly due to insufficient contrast of the heparin molecules with respect to the electron-dense metal NPs. The shape and dimensions of the NiO and Fe_3_O_4_ NPs can be observed in Figure 8C,D, respectively. The Co_3_O_4_ NP morphology appears quite different from the NiO and Fe_3_O_4_ NPs morphology; while the former shows globular shapes, the latter show much more crystalline shapes.

**Figure 9:**
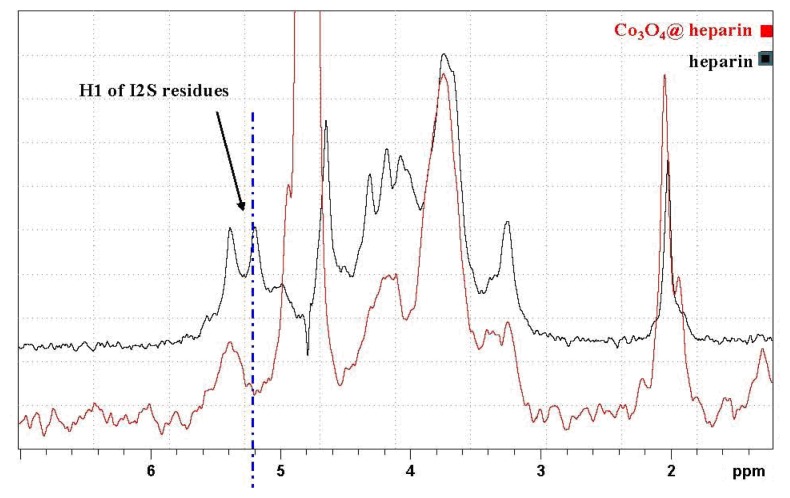
^1^H HR MAS NMR comparison of the heparin spectrum with that of Co_3_O_4_@heparin NP.

**Figure 10:**
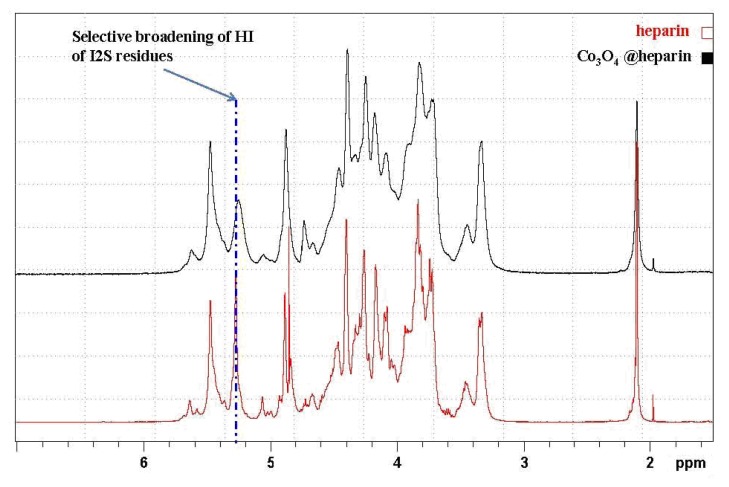
^1^H NMR comparison of the heparin solution spectrum with that of the Co_3_O_4_@heparin supernatant.

The formation of MO@heparin adducts has been confirmed by NMR spectroscopy. MO@heparin NPs described in Table 1 were analysed using ^1^H High-Resolution Magic Angle Spinning (HR MAS) NMR (Figure 9), which combines the typical advantages of solid- and liquid-state NMR techniques, and, when possible, by solution NMR spectrometry (Figure 10). With the mass ratio of MO@heparin components being in favour of the inorganic part, the solid-state ^13^C CP-MAS and MAS techniques were not of sufficient sensitivity to detect the polysaccharide. ^1^H HR-MAS NMR spectra should, in principle, permit the detection of the carbohydrate connected to the particle surface [48].

In our case, this detection was successful only for Co_3_O_4_@heparin NP. The lack of heparin detection for Fe_3_O_4_@heparin and NiO@heparin NPs can be explained by the drastic reduction of its mobility due to a very strong connection to the metal oxide surface. Starting from the assumption that the heparin NPs coating occurred in a heterogeneous phase, TEM results could be used to argue about HR-MAS NMR results. Figure 8 seems to suggest a relationship between the Fe_3_O_4_/NiO NPs morphology and the lack of heparin detection. The crystalline Fe_3_O_4_/NiO NPs could reduce the mobility of the coated highly charged heparin by forming ordered and rigid Fe_3_O_4_@heparin and NiO@heparin NPs. The interaction between the apparently amorphous Co_3_O_4_ NP and heparin could allow the heparin coating to maintain a certain grade of mobility.

In Figure 9, the HR-MAS spectrum of Co_3_O_4_@heparin NP suspended in D_2_O is compared with that of heparin alone. The selective disappearance of the anomeric signal (H1) of 2-*O*-sulphate iduronic acid (I2S) residues is evident and indicates their interaction with the surface of Co_3_O_4_.

To investigate this interaction in more detail, we used conventional NMR techniques to examine the supernatant of the Co_3_O_4_@heparin centrifugation isolating step, which contains some residual Co_3_O_4_@heparin NP and an excess of heparin (see the monodimensional 1D spectra presented in Figure 10). The same selective broadening of the uronic acid was observed upon introduction of paramagnetic species [49,50].

**Figure 11:**
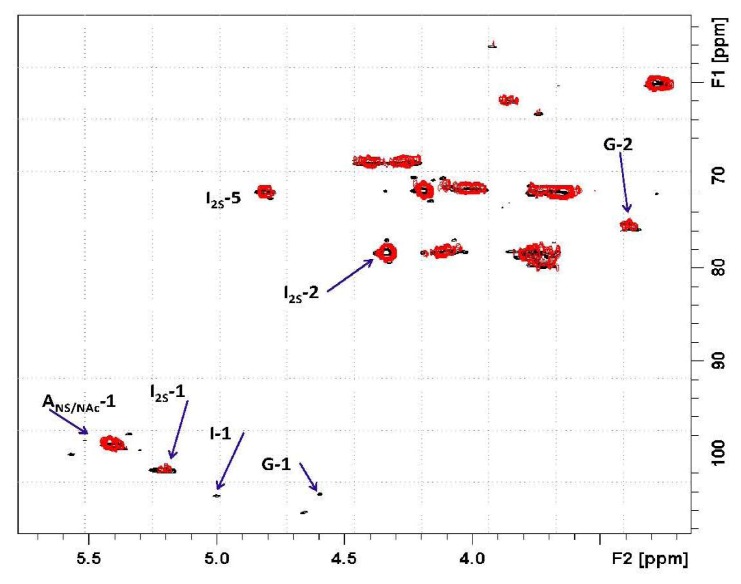
2D hetero-nuclear correlation NMR spectrum of the Co_3_O_4_@heparin supernatant.

**Figure 12:**
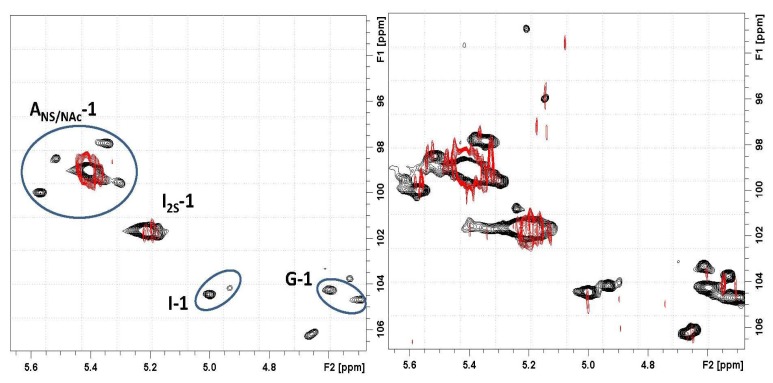
(**Left**) Expansion of the anomeric region of the 2D hetero-nuclear correlation NMR spectrum of the Co_3_O_4_@heparin supernatant ( 
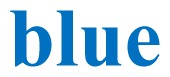
) overlapped with the mother heparin (black); (**Right**) Same expansion with signal intensities increased up to the noise level.

Based on the 2D heteronuclear correlation NMR spectrum of the Co_3_O_4_@heparin supernatant, the previous HR-MAS result was confirmed and additional details were observed (see Figures 11 and 12). Indeed, not only the I2S residues but also minor non-sulphated iduronic and glucuronic residues are involved in the interaction, indicating that all of the uronic acid residues are responsible for the electrostatic interaction.

Moreover, the signal of the proton linked to the carbon bearing the sulphate residue of I2S is unaffected, supporting the hypothesis that the site of heparin- Co_3_O_4_ NP interaction is the carboxyl group.

The influence of substitution pattern and cation binding on conformation of heparin in solution has been studied by NMR [51]. The I2S anomeric signal collapses because of metal ion interaction; a similar effect occurred for H5, the closest proton to the carboxyl moiety. For Co_3_O_4_@heparin NP, this phenomenon did not occur, as the unaffected I2S H5 signal remains evident in both the 1D and the 2D hetero-correlation spectra.

Whereas the FT-IR technique helped to visualise the MO@heparin NPs and the TB test quantified the heparin coating, the NMR technique investigations offered an original method of examining MO@heparin NPs. According to NMR technique results, we can argue that the core in Fe_3_O_4_@heparin and NiO@heparin NPs seems to immobilize the heparin shell, while the core in Co_3_O_4_@heparin NP has specific interactions with the heparin shell that anyway maintains its mobility.

## 3. Experimental Section

### 3.1. Synthesis of MO@heparin NPs (Core@Shell: MO@heparin)

Fe_3_O_4_ (CAS Number 1317-61-9), Co_3_O_4_ (CAS Number 1308-06-1), and NiO (CAS Number 1313-99-1) NPs (particle size <50 nm by TEM measurements, as described by supplier) were purchased from Sigma-Aldrich (St. Louis, USA) and used as received. Heparin, in the form of sodium salt, was provided by LDO Company (Trino Vercellese, Italy). In a general synthesis experiment, a suspension of NPs in distilled water (4 mg/mL) obtained by ultra-sonication for 5 min (Sonica 5300MH-Soltec, Milan, Italy) was transferred into a solution of heparin (40 mg/mL, pH 7 adjusted with 0.01 N NaOH). The mixture was stirred overnight (130–150 rpm, 20–25 °C, Julabo SW22). Co_3_O_4_@heparin and NiO@heparin NPs were separated by centrifugation (1 h, 6300*g*, Hettich Zentrifugen-Rotina 35 F, Tuttlingen, Germany), whereas Fe_3_O_4_@heparin NP was separated using a magnet (Ni–Cu–Ni Nickel plated, magnetisation N45). MO@heparin NPs were exhaustively washed with diethyl ether and then dried in air and in a conventional oven at 50 °C for 1 h.

Following the same reaction conditions and work up in water, Fe_3_O_4_@heparin NPs were prepared in a phosphate buffered saline (PBS) solution (0.137 M NaCl, 3 mM KCl, 0.01 M Na_2_HPO_4_, and 2 mM KH_2_PO_4_), in water with 1% *w*/*w* Tween 20^®^ (Sigma Aldrich, St. Louis, USA) as the surfactant and in PBS with 1% *w*/*w* Tween 20^®^. The pH value after the dissolution of heparin in PBS was 7.2.

Fe_3_O_4_@heparin NPs were also prepared in water by changing the heparin/Fe_3_O_4_ ratio. Six milliliter of a 40 mg/mL solution of Fe_3_O_4_ was added, after 5 min of ultra-sonication, to different volumes of a 40 mg/mL heparin solution at pH 7.

### 3.2. IR Characterisation of MO@heparin NPs

Heparin, the final MO@heparin NPs and the starting NPs were mixed with infrared grade KBr in a convenient proportion. The solid phase Fourier transform infrared (FT-IR) spectra were collected on a Bruker IFS 25.

### 3.3. Standard Curve for the Toluidine Blue (TB) Assay

**Figure 13:**
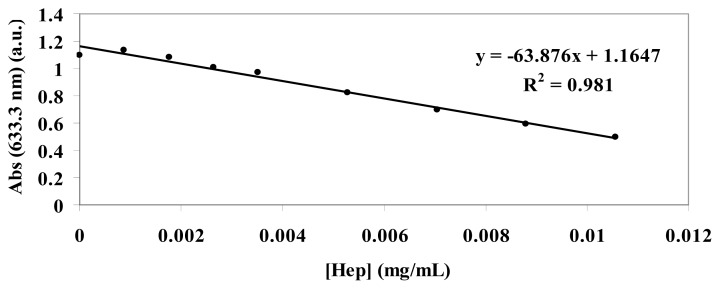
Standard curve showing the decrease in absorbance of a TB solution at 633 nm with increasing concentrations of heparin.

The standard curve of TB absorbance at 633 nm was constructed at increasing concentrations of heparin (range 0.01 mg/mL:0.0009 mg/mL) by mixing 1 mL of TB (0.04 mg/mL), 1 mL of water and 1 mL of different concentration heparin solutions (Figure 13). A second standard curve was obtained after extraction of each heparin:TB mixture with hexane. The two curves overlapped.

### 3.4. TB Assay Heparin Content in MO@heparin NPs and IR Characterisation of MO@heparin-TB Adducts

Suspensions obtained by mixing 1 mL of MO@heparin NPs or of bare NPs (0.05 mg/mL), 1 mL of TB (0.04 mg/mL) and 1 mL of water were stirred (30 min, 130–150 rpm, 25 °C, Julabo SW22) and centrifuged (20 min, 5000 rpm, 20 °C, Hettich Zentrifugen-Rotina 35 F). The supernatant TB blue solutions were UV analysed from 300 to 800 nm, and the absorbance at 633 nm was examined. The purple MO@heparin-TB adducts and the black bare NPs were dried under vacuum and investigated using IR, following the same procedure detailed for the MO@heparin NPs.

### 3.5. NMR Characterisation of Co_3_O_4_@heparin

The ^1^H High-Resolution Magic Angle Spinning (HR MAS) NMR spectra were recorded using a Bruker Avance 300 WB spectrometer (Karlsruhe, Germany) equipped with a 4-mm MAS probe. The samples were placed in a zirconium rotor, 4 mm in diameter and 21 mm high. Wet (D_2_O) sample analysis was run at a spin rate of 2500 Hz. The measurements were performed using a cpmg1ld standard sequence for water signal presaturation, under the following experimental parameters: D1 3 s, AQ 270 ms, P90 4 μs, NS 1024, T2 loop 16 ms, using tetramethylsilane as the reference.

Two-dimensional heteronuclear single quantum coherence (2D-HSQC) spectra were recorded at 25 °C on a Bruker Avance 500 spectrometer (Karlsruhe, Germany) equipped with a 5-mm TCI inverse probe under the following experimental conditions: carbon decoupling during acquisition, 320 increments of 32–64 scans. The polarisation transfer delay (*D* = 1/[2 × ^1^J_C–H_]) was established using ^1^J_C–H_ coupling values of 150 Hz. The matrix size 1 K × 512 was zero filled to 4 K × 2 K by applying a squared cosine function before Fourier transformation.

### 3.6. Dynamic Light Scattering (DLS) and Zeta Potential (ζ) of MO@heparin NPs and of Bare NPs

A 0.025 mg/mL solution of MO and MO@heparin NPs in water was prepared by immersing the solution in an ultrasonic bath for 1–5 s followed by centrifugation (Minispin, 1 min, 5000 rpm). The supernatant was immediately analysed in a folded capillary cell using DLS and Zetasizer Nano ZS, ZEN3600 from Malvern Instruments, at 25 °C and a scattering angle of 173°. The kinetic behaviour of Fe_3_O_4_@heparin in water was analysed based on DLS measurements at 37 °C using a quartz cuvette of 1 cm optic length Zetasizer Software 6.20 was used to analyse the field autocorrelation function in terms of the distribution of relaxation rates. The zeta potential size was automatically calculated from the electrophoretic mobility based on the Henry equation, UE = 2ɛζf(ka)/3η, where ζ is the zeta potential, UE is the measured electrophoretic velocity, η is the viscosity, ɛ is the dielectric constant and f(ka) is Henry’s function (adopted value: 1.5, referring to the Smoluchowski approximation). The Z-Average values obtained by DLS technique were calculated by the software as the average of ten measurements of the same suspension, each one obtained registering ten scans. The ζ values were calculated by the software as the average of six measurements of the same suspension.

The DLS and ζ measurements were also registered at increasing concentrations of free heparin by mixing 5 mL of Fe_3_O_4_@heparin (0.05 mg/mL) with different volumes of heparin (0.05 mg/mL) and adding water to reach a total volume of 10 mL. After 1 h., the solutions were centrifuged (Minispin, 5000 rpm, 1 min), and the supernatant was analysed at 25°C.

### 3.7. Transmission Electron Microscope (TEM) Characterisation of MO@heparin NPs and of Bare NPs

Five microliter drops of the different NP suspensions were deposited on 400 mesh copper grids coated with a formvar-carbon film (Società Italiana Chimici, Roma, Italy). After a few minutes, the excess liquid was removed using blotting paper. The grids were allowed to air dry at room temperature in a clean area. The samples were analysed using a Tecnai T12 TEM (FEI, Eindhoven, The Netherlands) operating at 120 kV.

## 4. Conclusions

Surface functionalisation of magnetic NPs is a necessary step for their application. In light of recent developments in nanomedicine, heparin-coated NPs may act as precious tools for cancer theranostics and surface functionalisation, with heparin being of particular interest because of its many biological activities. In this study, Co_3_O_4_, Fe_3_O_4_ and NiO NPs were directly conjugated to heparin, producing ultra-stable and intact heparin-coated NPs. The method is equally suitable for the three types of particles, and there are no significant differences in the heparin coating efficacy. The use of different analytic and characterisation techniques permitted us to describe in detail the heparin coated MO NPs. In particular, the heparin coating reduces the agglomeration of all the MO NPs in water and makes their surface highly negatively charged. Heparin coated MO NPs maintain the morphology of the bare NPs, but differ due to heparin coating mobility. The interaction with Fe_3_O_4_ and NiO reduce heparin mobility while the interaction with Co_3_O_4_ maintains heparin mobility as detected by NMR experiments.

## Figures and Tables

**Table 1:** Gravimetric and colorimetric data for heparin coating.

NP	Starting NP (mg)	MO@heparin weight increase (mg)	TB assay heparin coating (mg)
Co_3_O_4_	100	11	10–19
NiO	100	19	10–22
Fe_3_O_4_	100	5	10–15

**Table 2:** Heparin coating of Fe_3_O_4_@heparin NP prepared under different heparin/Fe_3_O_4_ ratio, determined by the colorimetric assay.

Starting Heparin (g/mL)	Starting NP (g/mL)	Heparin/NP *w*/*w*	TB assay heparin coating (mg)
0.033	0.003	11	15
0.028	0.010	2.8	9
0.022	0.016	1.4	2

**Table 3:** Z-A and PDI of MO@heparin NPs.

MO@heparin	Z-A (nm)	PDI
Co_3_O_4_@heparin	103	0.17
Fe_3_O_4_@heparin	92	0.17
NiO@heparin	93	0.23

**Table 4:** Bare and coated NP ζ.

NP	ζ (mV)	MO@heparin	ζ (mV)
Co_3_O_4_	−31	Co_3_O_4_@heparin	−70
Fe_3_O	−14	Fe_3_O_4_@heparin	−61
NiO	+19	NiO@heparin	−47

**Table 5:** Z-A, PDI and ζ of Fe_3_O_4_@heparin (0.025 mg/mL) in the presence of different amounts of free heparin.

heparin:Fe_3_O_4_@heparin (*w:w*)	Z-A (nm)	PDI	ζ (mV)
0:1	118	0.24	−54
0.1:1	112	0.26	−38
0,2:1	113	0.27	−70
0.4:1	118	0.24	−69
1:1	118	0.24	−73

**Table 6:** Comparison of coating, size and ζ potential values of Fe_3_O_4_@heparin obtained under different reaction conditions.

Reaction Conditions	Starting NP (mg)	Weight increase (mg)	TB assay heparin coating (mg)	Z-A (nm)	PDI	ζ Pot (mV)
Water + Tween 20^®^	100	5	9	93	0.25	−46
PBS	100	6	9	-	0.38	−47
PBS + Tween 20^®^	100	8	10	-	0.32	−49

## References

[1] Ghosh C.R., Paria S. (2012). Core/shell nanoparticles: Classes, properties, synthesis mechanisms, characterization, and applications. Chem. Rev.

[2] Janczak C.M., Aspinwall C.A. (2012). Composite nanoparticles: The best of two worlds. Anal. Bioanal. Chem.

[3] Katz E., Willner I. (2004). Integrated Nanoparticles-biomolecule hybrid systems: Synthesis, properties and applications. Angew. Chem. Int. Ed.

[4] Lu A.H., Salabas E.L., Schüth F. (2007). Magnetic nanoparticles: Synthesis, protection, functionalization and application. Angew. Chem. Int. Ed.

[5] De M., Ghosh P.S., Rotello V.M. (2008). Applications of nanoparticles in biology. Adv. Mater.

[6] Moyano D.F., Rotello V.M. (2011). Nano meets biology: Structure and function at the nanoparticle interface. Langmuir.

[7] Thanh N.T.K., Green L.A.W. (2010). Functionalisation of nanoparticles for biomedical applications. Nano Today.

[8] Lisi F., Falcaro P., Buso D., Hill A.J., Barr J.A., Crameri G., Nguyen T.-L., Wang L.-F., Mulvaney P. (2012). Rapid detection of hendra virus using magnetic particles and quantum dots. Adv. Health. Mater.

[9] Chen X., Gambhir S.S., Cheon J. (2011). Theranostic nanomedicine. Acc. Chem. Res.

[10] Cattaneo A.G., Gornati R., Sabbioni E., Chiriva-Internati M., Cobos E., Jenkins M.R., Bernardini G. (2010). Nanotechnology and human health: Risks and benefits. J. Appl. Toxicol.

[11] Garg H.G., Linhardt R.J., Hales C.A. (2005). Chemistry and Biology of Heparin and Heparan Sulfate.

[12] Hostettler N., Naggi A., Torri G., Isahai-Michaeli R., Casu B., Vlodavsky I., Borsig L. (2007). P-selectin- and heparanase-dependent antimetastatic activity of non-anticoagulant heparins. FASEB J.

[13] Bendas G., Lubor B. (2012). Cancer cell adhesion and metastasis: Selectins, integrins, and the inhibitory potential of heparins. Int. J. Cell. Biol..

[14] Casu B., Vlodavsky I., Sanderson R.D. (2007). Non-anticoagulant heparins and inhibition of cancer. Pathophysiol. Haemost. Thromb.

[15] Gupta A.K., Naregalkar R.R., Vaidya V.D., Gupta M. (2007). Recent advances on surface engineering of magnetic iron oxide nanoparticles and their biomedical applications. Nanomedicine.

[16] Mornet S., Vasseur S., Grasset F., Duguet E. (2004). Magnetic nanoparticle design for medical diagnosis and therapy. J. Mater. Chem.

[17] Don H., Sun X., Sun S. (2011). Monodisperse magnetic nanoparticles for theranostic applications. Acc. Chem. Res.

[18] Mahmoudi M., Simchi A., Milani A.S., Stroeve P. (2009). Cell toxicity of superparamagnetic iron oxide nanoparticles. J. Colloid Interf. Sci.

[19] Li Z., Kawashita M., Araki N., Mitsumori M., Hiraoka M., Doi M. (2010). Magnetite nanoparticles with high heating efficiencies for application in the hyperthermia of cancer. Mater. Sci. Eng. C.

[20] Munnier E., Cohen-Jonathan S., Herve K., Linassier C., Souce M., Dubois P., Chourpa I. (2011). Doxorubicin delivered to MCF-7 cancer cells by superparamagnetic iron oxide nanoparticles: Effects on subcellular distribution and cytotoxicity. J. Nanopart. Res.

[21] Kievit F.M., Zhang M. (2011). Surface engineering of iron oxide nanoparticles was studied for targeted cancer therapy. Acc. Chem. Res.

[22] Klostergaard J., Seeney C.E. (2012). Magnetic nanovectors for drug delivery. Nanomed. Nanotech. Biol. Med.

[23] Papis E., Gornati R., Prati M., Ponti J., Sabbioni E., Bernardini G. (2007). Gene expression in nanotoxicology research: Analysis by differential display in BALB3T3 fibroblasts exposed to cobalt particles and ions. Toxicol. Lett.

[24] Papis E., Rossi F., Raspanti M., Dalle-Donne I., Colombo G., Milzani A., Bernardini G., Gornati R. (2009). Engineered cobalt oxide nanoparticles readily enter cells. Toxicol. Lett.

[25] Sabbioni E., Fortaner S., Farina M., del Torchio R., Petrarca C., Bernardini G., Mariani-Costantini R., Perconti S., di Giampaolo L., Gornati R. (2012). Interaction with culture medium components, cellular uptake and intracellular distribution of cobalt nanoparticles, microparticles and ions in Balb/3T3 mouse fibroblasts. Nanotoxicology.

[26] Pan Y., Du X., Zhao F., Xu B. (2012). Magnetic nanoparticles for the manipulation of proteins and cells. Chem. Soc. Rev.

[27] Apátiga L.M., Castaňo V.M. (2006). Magnetic behavior of cobalt oxide films prepared by pulsed liquid injection chemical vapor deposition from a metal-organic precursor. Thin Solid Films.

[28] Ctistis G., Papaioanno E., Patok P., Gute J., Fumagalli P., Giersig M. (2009). Optical and magnetic properties of hexagonal arrays of subwavelength holes in optically thin cobalt films. Nano Lett.

[29] Salavati-Niasari M., Khansari A., Davar F. (2009). Synthesis and characterization of cobalt oxide nanoparticles by thermal treatment process. Inorg. Chim. Acta.

[30] Wolff A., Frese K., Wißbrock M., Eckstädt K., Ennen I., Hetaba W., Löffler S., Regtmeier A., Thomas P., Sewald N. (2012). Influence of the synthetic polypeptide c25-mms6 on cobalt ferrite nanoparticle formation. J. Nanopart. Res.

[31] Zhao J., Deng M., Zeng J., Huang Z., Yin G., Liao X., Gub J., Huang J. (2012). Preparation of Fe_3_O_4_ and CoFe_2_O_4_ nanoparticles with cellular compatibility via the histidine assistance. Colloid Surf. A.

[32] Moghaddam A.B., Ganjali M.R., Dinarvand R., Razavi T., Saboury A.A., Moosavi-Movahedi A.A., Norouzi P. (2008). Direct electrochemistry of cytochrome C on electrodeposited nickel oxide nanoparticles. J. Electroanal. Chem.

[33] Lee K.S., Lee I.S. (2008). Decoration of superparamagnetic iron oxide nanoparticles with Ni^2+^: Agent to bind and separate histidine-tagged proteins. Chem. Commun.

[34] Luo L., Li F., Zhu L., Ding Y., Zhang Z., Deng D., Lu B. (2013). Nonenzymatic glucose sensor based on nickel(II)oxide/ordered mesoporous carbon modified glassy carbon electrode. Colloid Surf. B.

[35] Tassa C., Shaw S.Y., Weissleder R. (2011). Dextran-coated iron oxide nanoparticles: A versatile platform for targeted molecular imaging, molecular diagnostics, and therapy. Acc. Chem. Res.

[36] Chertok B., Moffat B.A., David A.E., Yu F., Bergemann C., Ross B.D., Yang V.C. (2008). Iron oxide nanoparticles as a drug delivery vehicle for MRI monitored magnetic targeting of brain tumors. Biomaterials.

[37] Kemp M.M., Linhardt R.J. (2010). Heparin-based nanoparticles. Nanomed. Nanobiotechnol.

[38] Lee J., Jung M.J., Hwang Y.H., Lee Y.J., Lee S.S., Lee D.Y., Shin H. (2011). MRI of transplanted surface-labeled pancreatic islets with heparinized superparamagnetic iron oxide nanoparticles. Biomaterials.

[39] Hong R., Fischer N.O., Verma A., Goodman C.M., Emrick T., Rotello V.M. (2004). Control of protein structure and function through surface recognition by tailored nanoparticle scaffolds. J. Am. Chem. Soc..

[40] Irrgang J., Ksienczyk J., Lapiene V., Niemeyer C.M. (2009). Analysis of non-covalent bioconjugation of colloidal nanoparticles by means of atomic force microscopy and data clustering. ChemPhysChem.

[41] Medintz I.L., Uyeda H.T., Goldmann E.R., Mattoussi H. (2005). Quantum dot bioconjugates for imaging labelling and sensing. Nat. Mater.

[42] Clapp A.R., Medintz I.L., Mattoussi H. (2006). Förster resonance energy transfer investigations using quantum-dot fluorophores. ChemPhysChem.

[43] Khurshid H., Kim S.H., Bonder M.J., Colak L., Ali B., Shah S.I., Kiick K.L., Hadjipanayis G.C. (2009). Development of heparin-coated magnetic nanoparticles for targeted drug delivery applications. J. Appl. Phys.

[44] Smith P.K., Mallia A.K., Hermanson G.T. (1980). Colorimetric method for the assay of heparin content in immobilized heparin preparations. Anal. Biochem.

[45] Legrand L., Sagon G., Lecomte S., Chausse A., Messina R. (2001). A raman and infrared study of a new carbonate green rust obtained by electrochemical way. Corros. Sci.

[46] Ishikawa T., Ueno T., Yasukawa A., Kandori K., Nakayama T., Tsubota T. (2003). Influence of metal ions on the structure of poorly crystallized iron oxide rusts. Corros. Sci.

[47] Deraz N.M. (2008). Production and characterization of pure and doped copper ferrite nanoparticles. J. Anal. Appl. Pyrolysis.

[48] Simpson A.J., Kingery W.L., Shaw D.R., Spraul M., Humpfer E., Dvortsak P. (2001). The application of ^1^H HR-MAS NMR spectroscopy for the study of structures and associations of organic components at the solid-aqueous interface of a whole soil. Environ. Sci. Technol.

[49] Neville G.A., Mori F., Holme K.R., Perlin A.S. (1989). Monitoring the purity of pharmaceutical heparin preparations by high-field ^1^H-nuclear magnetic resonance spectroscopy. J. Pharm. Sci.

[50] Smernik R.J., Oades J.M. (1999). Effects of added paramagnetic ions on the ^13^C CP/MAS NMR spectrum of a de-ashed soil. Geoderma.

[51] Rudd T.R., Guimond S.E., Skidmore M.A., Duchesne L., Guerrini M., Torri G., Cosentino C., Brown A., Clarke D.T., Turnbull J.E. (2007). Influence of substitution pattern and cation binding on conformation and activity in heparin derivatives. Glycobiology.

